# Generalized perceptual adaptation to second-language speech: Variability, similarity, and intelligibilitya)

**DOI:** 10.1121/10.0020914

**Published:** 2023-09-12

**Authors:** Ann R. Bradlow, Adrianna M. Bassard, Ken A. Paller

**Affiliations:** 1 Department of Linguistics, Northwestern University, Evanston, Illinois 60208, USA; 2 Department of Psychology, Northwestern University, Evanston, Illinois 60208, USA

## Abstract

Recent work on perceptual learning for speech has suggested that while high-variability training typically results in generalization, low-variability exposure can sometimes be sufficient for cross-talker generalization. We tested predictions of a similarity-based account, according to which, generalization depends on training-test talker similarity rather than on exposure to variability. We compared perceptual adaptation to second-language (L2) speech following single- or multiple-talker training with a round-robin design in which four L2 English talkers from four different first-language (L1) backgrounds served as both training and test talkers. After exposure to 60 L2 English sentences in one training session, cross-talker/cross-accent generalization was possible (but not guaranteed) following either multiple- or single-talker training with variation across training-test talker pairings. Contrary to predictions of the similarity-based account, adaptation was not consistently better for identical than for mismatched training-test talker pairings, and generalization patterns were asymmetrical across training-test talker pairs. Acoustic analyses also revealed a dissociation between phonetic similarity and cross-talker/cross-accent generalization. Notably, variation in adaptation and generalization related to variation in training phase intelligibility. Together with prior evidence, these data suggest that perceptual learning for speech may benefit from some combination of exposure to talker variability, training-test similarity, and high training phase intelligibility.

## I. INTRODUCTION

Word recognition accuracy rates by both human and computer listeners often drop substantially when presented with speech by second-language (L2) talkers.^1–9^ However, rapid and substantial improvements in L2 speech recognition have been widely demonstrated following training with L2 speech samples (see Ref. 10 for a recent and comprehensive review). A key challenge for understanding this remarkable flexibility in speech perception is to identify the training conditions that lead to optimal adaptation, that is, for perceptual adaptation that generalizes from a small set of initial exposure stimuli to improved recognition accuracy of novel stimuli produced by novel L2 talkers from both trained and novel accents. In addition to the benefits of enhancing speech communication across a language barrier for individuals and society, this research enterprise provides a window into the short- and long-term dynamics of the processes and representations that support language-comprehension constancy in the face of natural and pervasive speech variation.

Successful approaches to perceptual adaptation to L2 speech generally build on an understanding of L2 speech production as involving several confluent sources of systematic deviations from first language (L1) speech. First, individual differences in vocal tract anatomy and physiology combine with other learned patterns of speech and language production to yield talker-specific trait characteristics that remain consistent across both the L1 and L2 speech of an individual talker.^11–13^ Second, the acoustic characteristics of L2 (or “foreign-accented”) speech reflect interactions between the sound structures of the language being spoken and the talker's first language at various levels of linguistic structure (sub-segmental, segmental, syllabic, lexical, and phrasal). These talker-general/accent-specific L1–L2 interactions (see Ref. 14 for a review) naturally extend across groups of L2 talkers from the same L1 background and underlie the acoustic signature of a particular accent (e.g., French-accented English versus Japanese-accented English, or English-accented Spanish versus Italian-accented Spanish). Finally, several universal features associated with speech and language production in a non-dominant language are prominent in L2 speech (e.g., slower overall speaking rate and higher rates of syllable deletion), regardless of the language being spoken or the L1 of the talker.^15^ The high degree of systematicity of each of these confluent streams of influence on L2 speech production suggests that perceptual adaptation to L2 speech should generalize from a relatively limited set of training materials to novel materials by a trained talker (talker-specific adaptation), to novel materials by a novel talker from the same accent group (talker-general/accent-specific adaptation), and even to novel materials by a novel talker from a new accent group (talker-general/accent-general adaptation). Indeed, prior work has demonstrated increasingly expansive generalization, from talker-specific to talker-general/accent-specific to accent-general adaptation, in response to increasing variance along relevant dimensions in the training stimuli, specifically training on multiple talkers within an accent group for talker-general/accent-specific generalization^16,17^ and training on multiple talkers from different accents for accent-general adaptation.^18,19^ However, in view of a recent study showing cross-talker generalization following low-variability (i.e., single talker) training^20,21^ and another that found cross-accent generalization following one, but not other multiple-accent training conditions,^19^ the precise conditions that facilitate or constrain increasingly broad generalization remain open to debate and investigation.

One approach to perceptual learning for speech, the high-variability training approach, emphasizes exposure to variability during training as a means of promoting generalization of adaptation beyond the training stimuli. This approach was successfully applied to the notoriously difficult case of training Japanese listeners to identify English /r/ versus /l/^22,23^ and then extended to numerous cases of speech perception learning, including various novel phoneme contrasts, L2 vocabulary learning, as well as perceptual adaptation to L2 speech by L1 listeners (see Ref. 24 for a recent review of the theory and history behind this approach). While the high-variability training approach has generally been successful, recent studies have provided compelling evidence that high-variability exposure may, in fact, not be necessary for generalized learning of novel L2 contrasts^24^ or for adaptation to L2 speech.^20^ The crux of the evidence in these studies is the demonstration of roughly equivalent generalization of learning following high-variability training and some low-variability training conditions. In particular, for perceptual adaptation to L2 speech,^20^ directly compared high-variability (multiple-talker) and low-variability (single-talker) training conditions with a design that disentangled training format (single- versus multiple-talker) from the specific talkers presented at training and at test. This was achieved by rotating test and training talkers so that each of the four test talkers also served as a training talker in both single- and multiple-talker training formats. This design revealed substantial variation across the single-talker training conditions with some training-test talker pairs exhibiting as much cross-talker generalization as the multi-talker training condition.

This finding, together with other research,^19,21^ has led to the proposal that the crucial condition for cross-talker generalization of perceptual adaptation to L2 speech is training-test talker similarity. According to this proposed similarity-based account, generalization requires that the training stimuli provide the trainees with sufficient exposure to the phonetic properties of the test stimuli. Such exposure may come from one or more talkers in a multiple-talker training regimen. In the case of single-talker training, some training-test talker pairings will exhibit sufficient phonetic similarity to promote cross-talker generalization, while others will be too dissimilar for training with one talker to generalize to improved recognition of speech by the other talker without additional talker-specific exposure. Thus, according to this similarity-based account, the mechanisms that underlie cross-talker generalization, following single-talker training, are identical to those that underlie talker-specific adaptation, and the crucial conditions for generalized learning depend on the combination of training and test stimuli rather than on any feature of either the training or the test procedures (see Ref. 20 for a full explanation of this account in relation to perceptual adaptation to L2 speech). Importantly, this proposal suggests that exposure to variability *per se* is not crucial and that low-variability training may be sufficient for generalized learning. High-variability training may provide greater opportunities for training-test similarity; nevertheless, in either high- or low-variability training, the crucial element for generalization is sufficient training-test phonetic similarity.

Building on this work, the present study explored perceptual adaptation and generalization for L2 speech recognition with a round-robin design that like Xie *et al.*^20^ and Alexander and Nygaard,^19^ but unlike Baese-Berk *et al.*^18^ and Bradlow and Bent,^16^ disentangled training format (single-talker versus multiple-talker) from the specific talkers presented in the training and test phases. Like Xie *et al.*,^20^ this design has every talker serving as both a test talker and a training talker in both single-talker and multiple-talker training formats, allowing us to examine adaptation and generalization across all possible combinations of training and test talkers for the selected set of talkers. With this design, we test three predictions related to the proposed similarity-based account for highly generalized perceptual adaptation to L2 speech.

If high-variability (i.e., multiple-talker) training is not a necessary condition and low-variability (i.e., single-talker) training can be a sufficient condition to support highly generalized perceptual adaptation to L2 speech, then we should find that some training-test talker pair(s) promote generalization even following single-talker training. We use a round-robin design that allows us to directly compare 12 unique mismatched training-test talker pairings (i.e., pairings where the training and test talker are different individuals) with the expectation that we will find some pairs that support generalized perceptual adaption to L2 speech.

A strong interpretation of a similarity-based approach to perceptual adaptation to L2 speech predicts that talker-specific adaptation (i.e., same training and test talker) should consistently exceed cross-talker generalization since presumably, any given talker is more similar to themself than to any other talker. The round-robin design allows us to compare four different matched training-test talker pairings (i.e., pairings where the training and test talker are the same individual) with each other as well as with mismatched training-test talker pairings in the context of both single-talker and multiple-talker training formats.

Finally, because talker similarity is symmetrical (talker A is as similar to talker B as vice versa), the similarity-based approach predicts symmetrical patterns of generalization following single-talker training (training talker A with test talker B should yield similar improvement as training talker B with test talker A). The round-robin design allows us to test this prediction across the 12 mismatched training-test talker pairings. Asymmetrical training-test talker relations would implicate other factors beyond similarity that contribute to generalization of perceptual adaptation to L2 speech.

An important feature of the present study is that the stimuli were selected from a set of L2 English talkers who each came from a different L1 background. Thus, this study sought to extend prior tests of talker-independent/accent-specific generalization following both single-talker and multiple-talker training^20^ to the broadest level of generalization, that is, to accent-independent generalization. Prior tests of cross-accent generalization have yielded variable results. Bradlow and Bent^16^ included a post-training test with an untrained L2 English talker from an untrained accent, Slovak-accented English, following single-talker or multiple-talker training with Mandarin-accented English talkers. This study did not show any training-induced speech recognition improvement for the Slovak-accented talker, suggesting that the observed perceptual adaptation was limited to the trained accent—Mandarin-accented English. In a follow-up study, Baese-Berk *et al.*^18^ added a multiple-talker/multiple-accent training condition (five L2 English talkers from five different L1 backgrounds excluding Slovak-accented English) and found improved sentence recognition accuracy for the untrained talker/untrained accent—the Slovak-accented English talker. However, in both of these prior studies, the post-training tests always involved the same test talkers: one Mandarin-accented talker and one Slovak-accented talker. Thus, as noted by Xie *et al.*,^20^ it is impossible to know whether the lack of generalization following single-talker and single-accent training was due to the low-variability training procedure or to the specific test talkers used in these studies. The fragility of accent-general adaptation was also demonstrated by Alexander and Nygaard^19^ who found no improvement in word recognition accuracy on a multiple-talker test with Spanish-accented or Korean-accented English talkers following exposure to word-length stimuli by multiple speakers from the other accent (i.e., training with multiple Spanish-accented English talkers did not generalize to a test with multiple Korean-accented English talkers and vice versa). Interestingly, this study did find some hint of accent-general adaptation following training with a mixed accent group that had slightly higher levels of baseline intelligibility than either the Spanish-accented or Korean-accented group.

In the current study, we adopt a robust, round-robin design that like Alexander and Nygaard^19^ and Xie *et al.*^20^ avoided the limitation of a single test talker. Moreover, in a departure from Ref. 19, but in keeping with Refs. 16, 18, and 20, we use sentence-length stimuli. The rationale for using these longer stimuli is that, unlike word-length stimuli, sentences involve variations along multiple dimensions and at multiple time scales where the language-general properties of L2 speech are most likely to emerge (e.g., fewer syllables per second and less syllable-level reduction as shown in L2 speech in several languages by Ref. 15). It is important to note that since none of the individual L2 talkers in the set of L2 talkers included in the present study shared their first-language (L1) backgrounds, the present study did not address whether or how listeners form perceptual groups based on the L1 background of L2 talkers. That is, with only a single talker per L2 accent, the present data did not isolate talker-specific from accent-specific L2 speech features. Instead, this study addresses perceptual adaptation to L2 speech broadly defined without differentiation amongst accent groups.

## II. METHOD

The overall design for this study involved a training phase, followed by a test phase with an approximate 11 h delay between training and test [mean = 10 h = 53 min; standard error = 8 min]. This delay was in anticipation of a subsequent study (not reported here) involving sleep consolidation. Eight different training conditions (between-participants) were followed by an identical multiple-talker, multiple-accent sentence-in-noise recognition test. A round-robin arrangement of training and test talkers allowed us to compare perceptual adaptation to L2 English across various training-test talker combinations following both low-variability (single-talker) and high-variability (multiple-talker) training. The design also involved rotation of all talkers through both training and test talker roles so that we could probe training-test talker asymmetries. Importantly, the four talkers involved in the round robin all came from different L1 backgrounds (i.e., we had only one talker per L2 English “accent”). While this design feature provided an efficient data collection regimen for examination of both talker-specific and talker-independent/accent-independent adaptation to L2 speech, a single talker from each L1 background did not allow for direct replication of accent-dependent adaptation (i.e., adaptation to L2 features that are related to language-specific L1––L2 interactions) that have been demonstrated in prior work (e.g., Ref. 20).

### A. Participants

A total of 195 L1 American English listeners participated in this study. All participants lived in the United States, were between 18 and 35 yrs of age, self-reported as having no deficits in speech, language, or hearing, and as having normal or corrected-to-normal vision. Participants were recruited through an online experiment running resource (Prolific, https://www.prolific.co/) and paid for their participation. Those who were eligible for the study had the option of completing the training phase and were then invited back to complete the test phase. Participants were paid for their time promptly after each session. Only participants who completed both sessions were included in the final analyses. Informed consent was obtained electronically from all participants.

### B. Materials and procedure

A total of 300 sentence recordings were downloaded from the ALLSSTAR Corpus (https://groups.linguistics.northwestern.edu/speech_comm_group/allsstar2/#!/), an open-access corpus of L2 speech that includes both scripted and spontaneous recordings from over 100 L2 talkers from over 20 L1 backgrounds.^25^ For the present study, we compiled a set of simple sentences (e.g., “A towel is near the sink.”) from four L2 talkers from four different L1 backgrounds (75 sentences per talker), two males (L1 Brazilian Portuguese and L1 Spanish), and two females (L1 Farsi and L1 Turkish). These talkers were selected based on informal, subjective judgements by the authors as distinctly L2-accented with moderate-to-good comprehensibility. None of these talkers' recordings had been subjected to speech recognition testing prior to this study; therefore, no additional information about their overall intelligibility, subjective comprehensibility, or accentedness was available prior to the start of the study. In both the training and test phases, participants listened over headphones or earbuds to sentence recordings that had been digitally mixed with speech-shaped noise at a fixed signal-to-noise ratio (SNR) of 0 dB (i.e., the speech and noise were presented with equal loudness). This SNR ratio was selected based on prior studies in our laboratory with similar materials and procedures showing a wide range of intelligibility scores with ample room for improvement (e.g., Ref. 26). The sentences were presented one at a time with no possibility of repetition. Participants typed what they heard using the computer keyboard before advancing to the next sentence. No feedback was provided in either the training or test phase.

At test, participants were presented with 15 sentences by each of the four talkers (total = 60 sentences). The talker-sentence pairings were held constant across all training conditions while sentences presentations (i.e., trial orders) were randomized. Assignment of participants was counter-balanced across the eight training conditions: four single-talker and four multiple-talker, with 20–22 participants per condition. For comparison, Bradlow and Bent^16^ had 10 participants per training condition; Xie *et al.*^20^ had eight participants in each of their unique training-test talker pairings. All training conditions included the same 60 sentences, none of which also appeared in the test phase. An additional untrained control group (n = 27) took the multiple-talker test without any prior training phase.

In the single-talker training conditions, all 60 sentences were produced by one of the four talkers. Thus, at the test phase, listeners in single-talker training conditions encountered one trained talker and three novel talkers. In the multiple-talker training conditions, three of the four talkers produced 20 sentences each, while the fourth talker was excluded from the training set.^27^ Thus, at the test phase, listeners in multiple-talker training conditions encountered three trained talkers and one novel talker.

All sentence transcriptions from both the training and test phases were scored using an open-source automated scoring tool, Autoscore (http://autoscore.usu.edu/),^28^ which counts a sentence as either correctly (score = 1) or incorrectly (score = 0) recognized if, and only if, the transcription exactly matches the script from which the talkers read at the time of recording. Words that exhibited obvious spelling errors, or are homophones of the intended word, were counted as correct.

For analysis, proportional scores were log-odd transformed: ln((c + 0.5)/((n-c)+0.5)), where c is the number of words correctly recognized and n is the total number of words in the sentence. This transformation adjusts for the fact that the raw recognition scores can only take on values of 0 and 1. Separate single-talker and multiple-talker analyses were conducted because of the different balance of trained versus novel talkers encountered in the test following single-talker versus multiple-talker training (one trained and three novel for single-talker versus three trained and one novel for multiple-talker). Within each analysis, t-tests with Bonferroni correction for multiple comparisons (4 test talkers × 4 training conditions = 16) assessed improvement for each training talker-test talker pairing relative to the untrained control condition.

## III. RESULTS

Table I shows average sentence recognition accuracy across all participants in the untrained control condition, the four single-talker conditions, and the four multiple-talker training conditions. The speech recognition accuracy scores are broken down by talker within the multiple-talker test (columns). L1 codes for the single-talker training and test talkers are BRP = Brazilian Portuguese, FAR = Farsi, SPA = Spanish, and TUR = Turkish. For the single-talker training format, trials with matched training and test talkers are shown in bold. For the multiple-talker training format, condition codes indicate the talker that was excluded from the training set (all three of the other talkers were included): noBRP = Brazilian Portuguese excluded, noFAR = Farsi excluded, noSPA = Spanish excluded, and noTUR= Turkish excluded. Test trials with the novel talker (i.e., the talker excluded from the multiple-talker training set) are shown in bold. For all training conditions, the percentage point increase (or decrease) relative to the untrained control condition is also shown.

**TABLE I.** Average proportion of sentences correctly recognized. Percentage point increase (re controls): p < 0.05^c^ and p < 0.003^d^. For the single-talker training conditions, trials with matched training and test talkers are shown in bold. For the multiple-talker training conditions, the talker that was excluded from the training set is shown in bold.

	Test talker within the multiple-talker test (standard error in parentheses)			
	BRP^a^	FAR^a^	SPA^a^	TUR^a^
Control (n = 27)	0.784 (0.016)	0.722 (0.015)	0.712 (0.016)	0.545 (0.018)
Single-talker training conditions				
BRP (n = 21)	**0.843 (0.014) 5.9%^c^**	0.725 (0.018) 0.3%	0.727 (0.018) 1.5%	0.572 (0.02) 2.7%
FAR (n = 21)	0.886 (0.013) 10.2%^d^	**0.778 (0.016) 5.6%^c^**	0.767 (0.017) 5.5%^c^	0.599 (0.02) 5.4%^c^
SPA (n = 22)	0.852 (0.014) 6.8%^d^	0.723 (0.018) 0.1%	**0.726 (0.018) 1.4%**	0.581 (0.02) 3.6%
TUR (n = 20)	0.794 (0.017) 1.0%	0.676 (0.02) -4.6%	0.708 (0.018) -0.4%	**0.566 (0.021) 2.1%**
Multiple-talker training conditions				
noBRP^b^ (n = 22)	**0.854 (0.014) 7.0%** ^d^	0.76 (0.017) 3.8%	0.727 (0.018) 1.5%	0.626 (0.019) 8.1% ^d^
noFAR^b^ (n = 20)	0.827 (0.017) 4.3%^c^	**0.749 (0.018) 2.7%**	0.714 (0.019) 0.2%	0.609 (0.021) 6.4%^c^
noSPA^b^ (n = 21)	0.817 (0.017) 3.3%	0.739 (0.017) 1.7%	**0.727 (0.018) 1.5%**	0.566 (0.021) 2.1%
noTUR^b^ (n = 21)	0.878 (0.013) 9.4%^d^	0.794 (0.015) 7.2%^d^	0.778 (0.017) 6.6%^c^	**0.617 (0.019) 7.2%^c^**

^a^ L1 codes for the talkers are: BRP, Brazilian Portuguese; FAR, Farsi; SPA, Spanish; TUR, Turkish.

^b^ For the multiple-talker training conditions, the codes indicate the talker that was excluded, e.g., noBRP = training condition with stimuli produced by FAR, SPA, and TUR.

For the single-talker training conditions (top section of Table I), we see substantial variation across the 16 combinations of training and test talkers. Improvement relative to the untrained control condition emerged for four mismatched training-test talker pairings (i.e., pairings where the single training talker differed from the test talker), two at the Bonferroni corrected level of p < 0.003 (FAR and SPA training with the BRP test talker), and two at the less conservative level of p < 0.05 (FAR training with the SPA and TUR test talkers). In addition, two matched training-test talker pairing (i.e., where the single training talker was that same individual as the test talker) showed improvement over the untrained control condition at the p < 0.05 level (BRP training with the BRP test talker and FAR training with the FAR test talker). All other differences from baseline intelligibility failed to reach significance. The finding of highly generalized perceptual adaptation for some of the mismatched training-test talker pairings (i.e., pairings where the single training talker differed from the test talker) in the single-talker training format supports the claim that high-variability (i.e., multiple-talker) training is not necessary and low-variability (i.e., single-talker) training can be sufficient for highly generalized perceptual adaptation (Prediction 1).

With regard to talker-specific adaptation (Prediction 2), two of the four matched training-test talker pairings, BRP training talker with BRP test talker (BRP+BRP) and FAR training talker with FAR test talker (FAR+FAR) resulted in improvement (at the less conservative, uncorrected p < 0.05 level) over the baseline intelligibility level for that talker. For the other two matched training-test talker pairings, SPA training talker with SPA test talker (SPA+SPA) and TUR training talker with TUR test talker (TUR+TUR) there was no training-induced improvement over the baseline intelligibility level. Moreover, for two of the single-talker training conditions, FAR and SPA, the matched test talker trials were not the trials that showed the greatest percentage point improvement: FAR training led to greater improvement for the BRP test trials than for the FAR test trials, and SPA training led to greater improvement for the BRP and TUR test trials than for the SPA test trials. Thus, contrary to the prediction of a strict interpretation of the similarity-based account for generalized perceptual adaptation, we did not find evidence that talker-specific adaptation (i.e., the training-test talker pairing that represents the highest possible degree of training-test talker similarity) consistently exceeds cross-talker/cross-accent generalization.

Finally, contrary to the notion of training-test talker similarity as the driving mechanism for generalized perceptual adaptation (Prediction 3), the data showed asymmetries across several single-talker training conditions. Specifically, while FAR training generalized to the BRP test talker (p < 0.003), the SPA test talker (p < 0.05), and the TUR test talker (p < 0.05), none of these talkers (BRP, SPA or TUR), was an effective training talker for generalization to the FAR test talker. Similarly, while SPA training generalized to the BRP test talker, BRP training did not generalize to the SPA test talker.

For the multiple-talker training format (bottom section of Table I), we also see substantial variation across the 16 combinations of training condition and test talker. Pair-wise comparisons showed training-induced improvement at the Bonferroni corrected level of p < 0.003 for the BRP test talker following the multiple-talker training condition that excluded the BRP (noBRP) and TUR (noTUR) test talkers, as well as for the TUR test talker following the noBRP condition and the FAR test talker following noTUR training. In addition, several multiple-talker training conditions resulted in improved recognition accuracy at the less stringent level of p < 0.05: noFAR training for the BRP and TUR test trials, and noTUR training for the SPA and TUR test trials. Note that two of the four conditions with improvement, BRP test following noBRP training, and TUR test following noTUR training, are conditions that involve cross-talker/cross-accent generalization (noBRP training excluded the BRP talker, and noTUR training excluded the TUR talker). The other two multiple-talker training conditions, noFAR and noSPA, did not lead to cross-talker/cross-accent generalization. Thus, across all of the multiple-talker training conditions paired with all of the four test talkers, we do not find that multiple-talker training is consistently sufficient for highly generalized (i.e., cross-talker/cross-accent) perceptual adaptation following the relatively brief training procedure of the present study. We also note that since the design of this study involved a single talker for each accent (i.e., L1 background), the data do not address whether accent-specific adaptation can result from this multiple-accent training procedure.

Table II shows average percent improvement relative to the untrained control condition (i.e., test-control/control) for each training condition grouped by training format (single-talker versus multiple-talker) over the four test talkers. While these averages obscure the influence of specific training-test pairings (the main planned comparison in this study), the overall pattern reveals slightly higher average percent improvement following multiple-talker than single-talker training. However, the individual training conditions vary in the direction of change from single-talker to multiple-talker training format. Notably, the large increase in improvement from TUR ( –0.5%) to noTUR (11.1%) indicates that exclusion of the least effective single-talker training talker (TUR) from the multiple-talker training condition (noTUR) benefitted adaptation, while the decrease in improvement from FAR (9.6%) to noFAR (5.3%) indicates that exclusion of the most effective single-talker training talker (FAR) detracted from adaptation to L2 speech. This ranking in terms of training effectiveness largely reflects the ranking of baseline talker intelligibility as assessed from the untrained control condition (top row of Table I). Specifically, the talker associated with the lowest baseline intelligibility, TUR, was the least effective training talker in the single-talker training format and exclusion of this talker was associated with more effective training in multiple-talker training formats. In summary, the pattern of results shows considerable variation in training-induced perceptual adaptation following various training-test talker pairings in the context of both single-talker and multiple-talker training formats. The finding that some single-talker conditions resulted in significant cross-talker/cross-accent generalization indicates that high-variability exposure is not a necessary condition and low-variability training can be sufficient for highly generalized perceptual adaptation to L2 speech. This confirms the first prediction that we laid out in the introduction (Prediction 1). However, the present data did not support the second and third predictions which relate more directly to a strict interpretation of the similarity-based account for generalization. Specifically, contrary to Prediction 2, we did not find that talker-specific adaptation (the case with the highest possible degree of training-test talker similarity) consistently exceeded cross-talker/cross-accent generalization, and contrary to Prediction 3, we found asymmetrical generalization for certain training-test talker pairs, despite the fact that similarity is presumably a symmetrical relation. Interestingly, comparison of the single-talker and multiple-talker training formats, grouped by presence (single-talker condition) and absence (multiple-talker condition) of each of the four training talkers (Table II), offered some potential insight into training talker-specific factors, as opposed to pair-specific factors, which may contribute to variation in exposure-induced perceptual adaptation to L2 speech.

**TABLE II.** Average improvement relative to control, i.e., test-baseline/baseline.

Single-talker (%)		Multiple-talker (%)	
BRP	3.8	7.8	noBRP
FAR	9.6	5.3	noFAR
SPA	4.3	3.1	noSPA
TUR	−0.5	11.1	noTUR
Average	4.3	6.8	

## IV. ACOUSTIC ANALYSIS

To gain insight into the talker-specific acoustic characteristics that may have contributed to the observed pattern of adaptation and generalization, we conducted a series of acoustic analyses of the training and test stimuli. These analyses aimed to assess the extent to which acoustic similarity between training and test materials relates to variation in cross-talker/cross-accent generalization of perceptual adaptation as shown in Table I. We also examine inter-talker differences to see if there is any clear acoustic source for the variation across talkers in training effectiveness as shown in Table II. We approach this *post hoc* analysis with caution since, for sentence-length stimuli that were not designed to isolate specific acoustic cues, acoustic similarity/distance is a vague construct that defies simple objective measurement. A multitude of interacting acoustic parameters distinguish any two connected speech signals, and even with a standard set of sentences and controlled recording conditions, utterance-specific and situation-specific sources of intra-talker variability come in to play. This challenge has been noted in prior work, most pertinently by Alexander and Nygaard^19^ and Xie *et al.*,^20^ both of whom undertook acoustic analysis of training and test stimuli in their studies of perceptual adaptation to L2 speech. Neither of these studies found straightforward relations between their stimulus acoustics and patterns of perceptual improvement, an outcome that is consistent with decades of research demonstrating that the acoustic correlates of variation in speech intelligibility are highly complex and dynamic (e.g., Refs. 29–31). Nevertheless, in view of the relative novelty of the patterns of perceptual adaptation and generalization observed in the present study, we conducted a series of exploratory acoustic analyses of the stimulus set.

The design of this study involved separate sets of sentences for the training and test stimuli with no repetition across the training and test stimulus sets. We therefore selected five acoustic parameters that can be automatically measured and that can be meaningfully compared across different utterances. All acoustic analyses were conducted using the Praat program (https://www.fon.hum.uva.nl/praat/) for speech analysis.^32^ Measurements along the five acoustic parameters were extracted over eight sets of stimuli, four test sets, and four single-talker training sets. Each of the four test sets—TEST-PBR, TEST-FAR, TEST-SPA, and TEST-TUR—consisted of the 15 test sentences from a single talker. Because the identical multiple-talker test was administered following all training conditions and because no sentence was repeated over the course of the 60 test sentences, each talker's test set includes a different set of 15 sentences. In contrast, the four single-talker training sets— ST-PBR, ST-FAR, ST-SPA, and ST-TUR—included productions of the same 60 sentences by each talker.

The following analyses were applied to each of the eight sets of digital speech files.

### A. Articulation rate (acoustic syllables per second)

From each individual sentence file within each set, we obtained the number of acoustic syllables using a published Praat script^33^ that detects intensity peaks surrounded by intensity dips. The script's peak picking thresholds are designed to minimize the influence of non-speech sound bursts and to exclude peaks that occur during unvoiced portions of the signal. Articulation rate is expressed as the total number of peaks (acoustic syllables) divided by the duration of the sentence recording with major disfluencies (e.g., coughs) and silent pauses of at least 0.2 s excluded. For each of the eight stimulus sets, we calculated the average articulation rate over all sentences. Articulation rate variation has been shown to be a salient marker of language-independent talker-specificity in bilingual individuals^11^ as well as a highly consistent marker of L2 versus L1 speech across languages.^34,35^ Thus, articulation rate is a potentially important parameter for assessing similarity between two individual L2 talkers, and adaptation to the typically slow articulation rate of L2 speech should readily support cross-talker/cross-accent generalization.

### B. *F*0 mean and coefficient of variation (Hz)

The fundamental frequency at each acoustic syllable peak was extracted following application of the intensity peak picking script described above.^33^ The *F*0 mean and coefficient of variation were then calculated across these within-sentence values, and grand averages were calculated over all sentences in each of the eight stimulus sets to yield the *F*0 mean and coefficient of variation for each set. We take coefficient of variation (standard deviation/mean) as the measure of *F*0 variance rather than standard deviation to account for substantial variation in *F*0 means across the four talkers, presumably due to differences in larynx size. These *F*0 statistics are based exclusively on *F*0 values at acoustic syllable peaks to eliminate the influence of mis-tracked pitch points or intervals of glottal fry. Mean *F*0 reflects properties of a talker's vocal source and therefore, contributes to perceived talker similarity. *F*0 variation provides some indication of general effort (the greater the *F*0 range, the greater the effort) which may also serve as a parameter of similarity between talkers.

### C. Vowel dispersion (Bark)

Average vowel dispersion for each of the eight sets of sentence stimuli was determined from the Euclidian distance of each individual vowel from the average *F*1/*F*2 for the given stimulus set. Following initial processing with the Praat script that identified acoustic syllables (intensity peaks) and *F*0 frequencies,^33^ all individual sentence files within each of the eight sets were concatenated to yield eight single-talker, multiple-sentence files (digital speech files plus associated Praat textgrids marking the acoustic syllable peak locations). Then, *F*1 and *F*2 frequencies at each acoustic syllable peak were extracted, and the mean *F*1 and *F*2 frequencies were calculated across the full set of concatenated files to yield the location of the vowel space centroid for that set. Each vowel's distance from the centroid was then calculated using the formula in Eq. (1), where D is each vowel's distance from the vowel space center, ${F1}_{v}$ and ${F2}_{v}$ are the vowel's *F*1 and *F*2 values, respectively, and ${F1}_{x}$ and ${F2}_{x}$ are the centroid F1 and F2 values, respectively. Finally, the average vowel dispersion for the set was calculated as the average Euclidian distance across all vowels:

$$D=\sqrt{{\left({F1}_{v}-{F1}_{x}\right)}^{2}+{\left({F2}_{v}-{F2}_{x}\right)}^{2}}.$$ (1)

Vowel space dispersion indexes articulatory precision and effort with greater dispersion indicating more extreme tongue positioning for individual vowel articulations. The relatively crowded vowel inventory of English is challenging for L2 speakers from various L1 backgrounds. Thus, while language-specific L1–L2 vowel space interactions are often salient markers of a specific L2 English accent, adaptation to vowel space deviation is likely an important accent-general L2 speech adaptation strategy. Moreover, like *F*0 range, vowel dispersion can be considered as a proxy for articulatory effort and precision in general. These properties motivate vowel dispersion as a parameter of similarity between talker pairs.

### D. Syllable reduction rate

For each of the eight sets of sentences, the syllable reduction rate was calculated as the total number of acoustic syllables across the full set of concatenated sentences (as determined from the automatic intensity peak picking script described above) as a proportion of the number of canonical, orthographic syllables across all words in the texts of the sentence set (# acoustic syllables / # orthographic syllables). The number of orthographic syllables was determined using an English syllable counting function implemented in R (https://www.r-project.org/),^36^ which counts orthographic syllables in a given text, based roughly on the number of orthographic vowels with adjustments for common orthographic deviations from the one-vowel-one-syllable rule. Syllable reduction rate has been shown to distinguish L1 from L2 speech across various languages with L2 speech in English, French, and Spanish all exhibiting less syllable reduction than their L1 counterparts.^15^ This difference likely stems from less fluency with the typical reduction processes that characterize connected speech production and leads to L2 speech typically having lower information density and therefore, lower communicative efficiency than L1 speech (more syllables produced for a given utterance). Therefore, as a salient language-general feature of L2 speech, syllable reduction rate is a possible parameter of generalized adaptation to L2 speech as well as of L2 talker similarity.

Table III shows grand average values along each of the five acoustic parameters for the four sets of test stimuli (TEST-BRP, TEST-FAR, TEST-SPA, and TEST-TUR) and the four sets of single-talker training stimuli (ST-BRP, ST-FRA, ST-SPA, and ST-TUR). To assess similarity on the basis of a combination of acoustic parameters, we calculated the Euclidian distance between the feature vectors defined by the five acoustic parameters for each pair of training and test stimuli. We focus on Euclidian, rather than cosine distance, because our central concern is for variation in distance between two vectors, rather than variation in the orientation of the two vectors (which does not take into consideration how far they extend into the vector space).

**TABLE III.** Grand average values of acoustic measurements for test and single-talker training stimuli.^a^

	TEST-BRP (n = 60)	TEST-FAR (n = 60)	TEST-SPA (n = 60)	TEST-TUR (n = 60)	ST-BRP (n = 15)	ST-FAR (n = 15)	ST-SPA (n = 15)	ST-TUR (n = 15)
Articulation rate (syllables/second)	3.27	3.31	3.23	3.33	3.15	3.15	3.07	3.31
*F*0 variance (coefficient of variation, Hz)	0.09	0.18	0.16	0.12	0.13	0.15	0.19	0.11
Vowel dispersion (Bark)	2.83	3.09	2.56	3.05	3.15	3.03	2.8	3.05
Syllable reduction rate	0.79	0.77	0.81	0.75	0.82	0.78	0.82	0.75
*F*0 mean (Hz)	128.68	173.85	125.28	226.65	131.62	174.11	126.68	220.61

^a^ The number of sentences for each stimulus set shown in the column headings.

Table IV lists the Euclidian distances for each training-test talker pairing, along with the change in speech recognition accuracy for that pairing, relative to the untrained control condition. Values are shown for each of the 16 single-talker training-test conditions in descending order of speech recognition difference (percentage points). Euclidian distances are shown for both the five-feature vector space as well as for a four-feature vector space that excludes the *F*0 mean acoustic parameter as one of the vector features. The rationale for excluding *F*0 mean is that this feature reflects variation in talker gender (the male talkers, BRP and SPA, both have substantially lower *F*0 means than the female talkers, FAR and TUR) and therefore, this group-level *F*0 mean difference may overwhelm other individual-level phonetic similarity relations amongst the four talkers. Correlations between the Euclidian distance and training-induced change in speech recognition accuracy for each of the 16 training-test talker combinations in the five-feature and four-feature vector spaces were both non-significant. This indicates that, in a vector space with equal weightings for the parameters included in this analysis, talker similarity along these dimensions does not account for variation in cross-talker/cross-accent generalization of perceptual adaptation to L2 speech.^37^

**TABLE IV.** Recognition improvement and acoustic distances for all training-test talker pairs.^a^

Talker					1-Feature acoustic differences (train-test)				
Train	Test	Speech recognition differences (% points)	Five-feature Euclidian distance	Four-feature Euclidian distance	Articulation rate	*F*0 mean	*F*0 range	Vowel dispersion	Syllable reduction
FAR	BRP	10.20	45.437	0.241	−0.12	45.44	0.06	0.20	−0.01
SPA	BRP	6.80	2.011	0.231	−0.20	−2.00	0.10	−0.04	0.03
BRP	BRP	5.90	2.959	0.341	−0.12	2.94	0.04	0.32	0.02
FAR	FAR	5.60	0.317	0.178	−0.17	0.26	−0.03	−0.05	0.01
FAR	SPA	5.50	48.838	0.483	−0.08	48.84	−0.01	0.48	−0.03
FAR	TUR	5.40	52.532	0.187	−0.18	−52.53	0.03	−0.02	0.02
SPA	TUR	3.60	99.966	0.374	−0.26	−99.97	0.07	−0.25	0.07
BRP	TUR	2.70	95.029	0.217	−0.18	−95.03	0.01	0.10	0.06
TUR	TUR	2.10	6.036	0.026	−0.02	−6.04	−0.01	0.00	−0.01
BRP	SPA	1.50	6.367	0.598	−0.08	6.34	−0.03	0.59	0.01
SPA	SPA	1.40	1.432	0.292	−0.16	1.40	0.04	0.24	0.02
TUR	BRP	1.00	91.932	0.227	0.04	91.93	0.02	0.22	−0.05
BRP	FAR	0.30	42.235	0.189	−0.17	−42.23	−0.05	0.06	0.05
SPA	FAR	0.10	47.173	0.380	−0.24	−47.17	0.01	−0.29	0.05
TUR	SPA	−0.40	95.333	0.508	0.08	95.33	−0.05	0.50	−0.06
TUR	FAR	−4.60	46.758	0.084	0.00	46.76	−0.07	−0.03	−0.03

^a^ Rows sorted in descending order of speech recognition difference.

Table IV also lists the average training-test differences along each of the five acoustic parameters. For each parameter, a positive difference indicates that the value for the training set exceeded the value for the test set. If training-test talker similarity along a given parameter facilitates generalized perceptual adaptation, then we should find a negative correlation between the acoustic difference scores and speech recognition accuracy improvement (greater perceptual adaptation for smaller training-test difference). Only one of the five acoustic parameters showed a significant correlation between speech recognition improvement and acoustic difference from training to test. Specifically, change in *F*0 variation (*F*0 coefficient of variation in Hertz) was positively correlated (Pearson R = 0.67, p < 0.005) with change in speech recognition accuracy: a greater increase in *F*0 variance from training to test (i.e., when the *F*0 range within sentences was greater for the training stimuli than for the test stimuli) was associated with greater improvement in speech recognition accuracy from training to test. All other correlations between speech recognition improvement and acoustic difference along the individual acoustic parameters were non-significant.

Finally, with regard to inter-talker acoustic differences in relation to variation in training effectiveness, the values in Table III indicate that talker TUR is a notable outlier along most acoustic dimensions as well as in terms of baseline intelligibility. Specifically, TUR's baseline intelligibility falls approximately 20 percentage points below the other three talkers' baseline intelligibility scores (54.5% versus 71.2%, 72.2%, and 78.4%), and the ST-TUR stimulus set is an outlier in terms of speech rate (fastest), *F*0 variation (lowest), syllable reduction (lowest), and *F*0 mean (highest).

Overall, based on the *post hoc* and relatively limited acoustic analysis, we find substantial acoustic variation across the various training and test stimulus sets. However, we did not find support for the claim that acoustic similarity between training and test stimuli promotes generalized perceptual adaptation to L2 speech. At that same time, we cannot dismiss the possibility that other acoustic dimensions of similarity may show a tighter link between training-test similarity and cross-talker/cross-accent generalization than the current analyses.

## V. DISCUSSION

Motivated by recent demonstrations of cross-talker generalization of perceptual adaptation to L2 speech, following both low-variability and high-variability training,^20^ this study leveraged the systematic variation of L2 speech to probe the limits of such generalization and to test predictions of the similarity-based account of highly generalized perceptual learning for speech. Using a round-robin design with numerous training-test talker pairings, the results of the present study confirmed that, for some training-test talker pairs, single-talker/low-variability training with L2 speech recognition can be sufficient for cross-talker/cross-accent generalization. The data also showed that multiple-talker/high-variability training does not invariably lead to cross-talker/cross-accent generalization. This result adds to the growing body of data suggesting that exposure to talker variation *per se* may not be a necessary condition for generalized perceptual learning for speech. Instead, exposure to limited stimulus variation as produced by a single talker during training may be sufficient for perceptual learning to generalize beyond the training talker to a novel test talker. However, identifying the critical conditions that result in generalized learning remains a challenge.

The finding of cross-talker/cross-accent generalization for some, but not all, training-test talker pairs is consistent with the similarity-based account, according to which cross-talker generalization depends on sufficient overlap between training and test items in terms of the mapping from acoustic cues to linguistic categories. As noted by Xie *et al.*,^20^ quantification of acoustic similarity remains a vague and elusive construct for connected speech samples where variation is extensive along multiple phonetic parameters and at multiple time scales (segments, words, phrases, etc.). Indeed, our acoustic analysis, based on five global parameters, did not find any relation between training-test talker acoustic similarity and extent of cross-talker generalization of perceptual adaptation. It remains possible that other parameters might reveal a positive relationship between acoustic–phonetic similarity of training-test talker pairs and cross-talker generalization; nevertheless, in the absence of conclusive acoustic data regarding training-test talker similarity, we addressed two other predictions of the similarity-based account.

First, a narrow interpretation of the similarity-based account predicts a training-induced speech recognition accuracy advantage for matched training and test talker pairs (i.e., talker-specific adaptation) since each talker is presumably most similar to themself. However, contrary to this prediction, the present data did not show a consistent talker-specific training advantage. Instead, of the four matched single-talker conditions (i.e., training talker = test talker), only two showed improvement at the less stringent p < 0.05 level from the untrained control condition, and the conditions with the greatest amount of improvement were conditions with mismatched training and test talkers. The absence of significant talker-specific adaptation in the present study should be viewed in the context of the finding of cross-talker/cross-accent adaptation for some training-test talker pairings in the single-talker training conditions. In particular, the lack of significant talker-specific adaptation for BRP stands in contrast to the significant improvement observed for that test talker, BRP, following training with FAR and SPA. Under the assumption that cross-talker generalization requires more extensive adaptation than talker-specific adaptation, the lack of talker-specific adaptation for this talker, BRP, is surprising. It remains unclear what aspects of BRP's productions of the training stimuli versus FAR's and SPA's productions of those same sentences might account for this pattern. While these results do not provide positive evidence in favor of the similarity-based account, they also do not provide strong counter-evidence because it remains possible that for most of the training-test talker pairs, the acoustic–phonetic differences across the training and test sentence sets overwhelmed any discernible talker-specificity. Recall that, while the training sentences were identical across all training conditions, each of the four test talkers' post-test intelligibility scores were based on a different set of test sentences because the multiple-talker test necessarily involved no repeated sentences. Thus, it is possible that a larger set of sentences for both training and testing would allow for the emergence of a stronger talker-specificity advantage, as predicted by the similarity-based account. Moreover, relatively wide and irregular within-talker phonetic variation is likely to inhibit both talker-specific and talker-general adaptation; thus, the lack of unequivocal talker-specific adaptation for all talkers in the present dataset should be interpreted cautiously with regard to predictions of the similarity-based account.

Second, the round-robin design of the present study allowed us to compare generalization of perceptual adaptation within training-test talker pairs across conditions with reversed roles for each talker. Since similarity is a symmetrical relation, the similarity-based account predicts symmetrical adaptation and generalization patterns within pairs of talkers. However, the data from this study found asymmetrical adaptation and generalization: the cases of significant improvement in speech recognition accuracy for a given training-test talker pairing did not show significant improvement for the conditions where the talkers reversed roles. This finding strongly suggests that some factor, other than similarity, plays an important role in establishing the necessary conditions for generalized perceptual adaptation to L2 speech.

The patterns emerging in this dataset suggest a possible role for intelligibility in terms of sentence recognition accuracy. Specifically, the talker associated with the lowest baseline intelligibility, TUR, was the least effective training talker across the four single-talker training conditions and the most effective multiple-talker training condition was the condition that excluded this talker. However, variation in training talker intelligibility cannot account for all aspects of the present dataset since, for example, similar baseline intelligibility was associated with SPA and FAR. Yet, they diverged in their effectiveness as single-talker training talkers, with FAR as the most effective training talker across the group of four talkers and SPA as close to average. Moreover, it is important to note that the training and test conditions in this study were not constructed to vary systematically in intelligibility. After completion of this study, we began data collection for an unrelated large-scale study of L2 English speech recognition which includes the four talkers in the present study. Despite several differences in the overall experimental setup, data from a comparable condition with L1 English listeners, 0 dB SNR with broadband noise, and mono-clausal sentence stimuli, showed a broadly consistent ranking of the four talkers, with BRP and TUR as the highest and lowest intelligibility talkers, respectively. Average and standard errors for sentence recognition accuracy rate across the four talkers in this subsequent study were: BRP 0.843 (0.02), SPA 0.706 (0.026), FAR 0.605 (0.028), and TUR 0.335 (0.028) (for comparison with the top row of Table I). These data provide independent confirmation that TUR is a low-intelligibility outlier in this group of four talkers.

However, prior research has provided equivocal support for the proposal that relatively low baseline intelligibility inhibits rapid perceptual adaptation to L2 speech. Bradlow and Bent^16^ found substantially slower improvement in sentence recognition accuracy by L1 English listeners from the first to the fourth quartile of trials within single-talker conditions for relatively low intelligibility L2 English talkers compared to relatively high intelligibility L2 talkers. Specifically, while the ceiling level of performance was reached by the second quartile of trials for the condition with the highest intelligibility talker, it took three quartiles worth of trials before any significant improvement was achieved for the lowest intelligibility talker. While this experiment did not test generalization to new stimuli or new talkers, it does provide some hint that variation in baseline intelligibility may play some role in the processes that underlie perceptual adjustments to L2 speech. Tzeng *et al.*^38^ directly investigated the role of intelligibility for generalized perceptual adaptation to L2 speech by comparing various orderings of high- and low-intelligibility training talkers within a multiple-talker training procedure, e.g., two low-intelligibility talkers followed by two high-intelligibility talkers in one condition compared to another condition with the reversed order of training talkers. The results of this study showed no impact on speech recognition accuracy in the post-training test on the order of training stimuli.

While it remains for future research to determine conclusively whether, when, and how intelligibility might contribute to generalized perceptual adaptation to L2 speech, it is worth noting that a role for intelligibility of training materials would be highly consistent with research on lexically guided perceptual learning for speech^39,40^ and many others. This prolific line of research has demonstrated a crucial role for lexical knowledge in exposure-induced recalibration of phonetic category boundaries. Recognition of meaningful sentences in L2 speech differs substantially from phonetic categorization along specific acoustic dimensions because it involves variations along a multitude of interacting phonetic dimensions and requires integration of phonetic processing with morphological, lexical, syntactic, semantic, and pragmatic knowledge. Nevertheless, we can hypothesize that if word recognition accuracy during training is too low (as might be the case with a low-intelligibility training talker), then lexical knowledge may not be available to guide the mapping of phonetic variation to linguistically meaningful categories, and consequently, adaptation to L2 speech may be constrained for both talker-specific and talker/accent-general adaptation. Conversely, if word recognition accuracy during training is high, then lexically guided recalibration of phonetic categories can presumably proceed relatively swiftly.

Table V shows sentence recognition accuracy scores from the training phase in each of the four single-talker training conditions. Scores for each quartile of trials (15 sentences/quartile) are shown. It is clear from these data that even in the final quartile of trials, participants in the TUR single-talker training phase were barely surpassing 50% recognition accuracy. For all other conditions, by the end of the training phase, participants were scoring above 75% correct recognition. These training phase data are consistent with the claim that TUR was an ineffective training talker due to consistently low recognition accuracy for this talker's speech during the training phase, thereby inhibiting lexically guided phonetic adjustment. It remains for future research to explicitly test this speculation with stimuli selected based on independent prior assessment of training phase intelligibility. Note, in particular, that intelligibility is a property of an interaction and intelligibility variation depends on a host of factors, including but not limited to, talker-dependent properties, transmission channel characteristics, and the speech, hearing, and language profile of the listener. Variation in each of these factors will result in variation in speech recognition accuracy (i.e., intelligibility). Therefore, if training phase intelligibility is a driving factor for generalized perceptual adaptation to L2 speech, then systematic variation along any of these factors—talker, communicative situation, and listener—should result in systematic variation in adaptation and generalization. Testing this claim for talker-specific, talker-general, as well as accent-general adaptation, is an important direction for future work. Of particular interest is the extent and manner in which lexically guided phonetic learning may interact with availability of other sources of linguistic and/or indexical guidance. For example, listeners may leverage prior experience with talkers from particular accent groups to adjust to novel talkers whose speech patterns resemble those of a previously encountered accent group. In other words, lexical guidance and perceived similarity at either the individual or group level, may conspire to promote highly generalized perceptual adaptation to L2 speech.

**TABLE V.** Average sentence recognition accuracy during the single-talker training phases with each talker by quartile of trials (15 sentences/quartile). Standard error is shown in parentheses.

	Single-talker training condition			
Quartile	PBR	FAR	SPA	TUR
1	0.776 (0.012)	0.658 (0.013)	0.738 (0.012)	0.417 (0.014)
2	0.846 (0.010)	0.710 (0.012)	0.792 (0.011)	0.476 (0.014)
3	0.847 (0.010)	0.741 (0.012)	0.797 (0.010)	0.490 (0.014)
4	0.860 (0.009)	0.754 (0.012)	0.784 (0.011)	0.504 (0.015)

This study aimed to advance our understanding of the exposure conditions that promote or constrain perceptual adaptation to L2 speech. We focused on the role of talker variation; however, it is important to also remain cognizant of other factors that may play into the exposure-induced adaptation that we observed. In particular, our design did not isolate adaptation to the phonetics of L2 English speech from adaptation to the task of speech-in-noise recognition. Prior work (e.g., Ref. 16) showed task adaptation for a trained control condition, relative to an untrained control condition where the trained control involved exposure to L1 English speech in the same noise condition as the test conditions. It is, therefore, important to note that the observed adaptation is most likely a combination of adaptation to the task of L2 speech-in-noise recognition and adaptation to the L2 speech samples. Nevertheless, the key comparisons for the present study were across training conditions. The observed variation across conditions strongly indicates that adaptation to the task cannot fully account for the data since all conditions offered identical opportunities for task adaptation; differences across training conditions must stem from differences in the training and/or test talkers.

## VI. CONCLUSIONS

Exposure to variability during training is not *necessary* and does not guarantee generalized perceptual adaptation to L2 speech. This conclusion is supported by the finding that not all multiple-talker and some single-talker training conditions resulted in significant cross-talker/cross-accent generalization, indicating that, for some training-test talker pairings, single-talker exposure is *sufficient* for generalization.

Training-test talker similarity cannot fully account for cross-talker/cross-accent generalization patterns. Contrary to predictions from a strict interpretation of a similarity-based account, we did not find that talker-specific adaptation consistently exceeded (or was at least equivalent) to talker-general adaptation. Furthermore, we found asymmetrical patterns of cross-talker generalization even though talker similarity is a symmetrical relation. Acoustic analyses also did not support a tight relation between acoustic similarity and cross-talker generalization of perceptual adaptation. Thus, our data do not support a narrow interpretation of the similarity-based account; however, it remains highly plausible that generalized adaptation to L2 speech benefits from phonetic similarity between the training and test stimuli at multiple levels of structure include both voice- and articulation-related properties.

Low intelligibility during training may inhibit perceptual adaptation to L2 speech. This conclusion is based on the finding that the least effective single-talker training condition involved the talker with the lowest baseline intelligibility, as well as with the lowest training phase intelligibility, and exclusion of this talker increased effectiveness of the multiple-talker training format. This unplanned finding suggests a role for training phase intelligibility in promoting highly generalized perceptual adaptation to L2 speech. In particular, it is possible that without sufficient word recognition accuracy during training, lexically guided phonetic category adjustment is constrained enough to prevent adaptation and generalization.

Taken together, these conclusions suggest that some combination of exposure to variability, training-test talker similarity, and training talker intelligibility may be the pathway to optimal improvement in L2 speech recognition accuracy. More generally, the fact that exposure to just 60 sentences in L2 English can result in improved recognition of novel sentences by novel talkers highlights the dynamic nature of speech communication between interlocutors from different language backgrounds.

## Data Availability

The stimuli for the study are stored on Speechbox,^41^ an openly available web-based system developed and maintained in the Linguistics Department at Northwestern University for managing and providing access to digital speech corpora. The speech recognition and acoustic analysis data gathered for this study are openly available on OSF.^42,43^
